# Supplementary figures and images for: Notoginsenoside R1 attenuates brain injury in rats with traumatic brain injury: Possible mediation of apoptosis via ERK1/2 signaling pathway
Source: PLoS One. 2023 Dec 18;18(12):e0295903. doi: 10.1371/journal.pone.0295903 (PMC10727368; doi:10.1371/journal.pone.0295903)

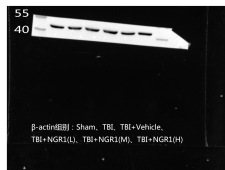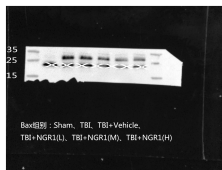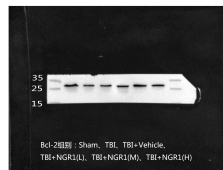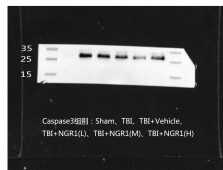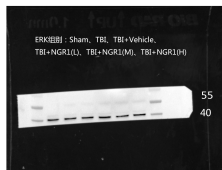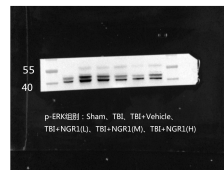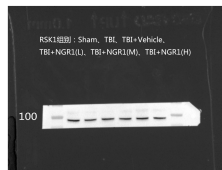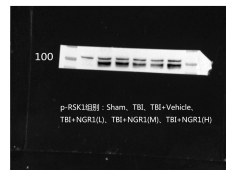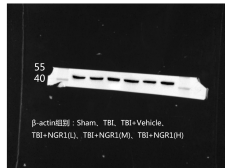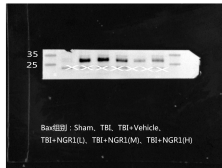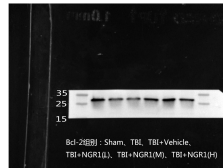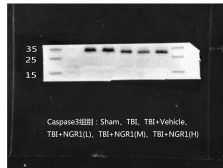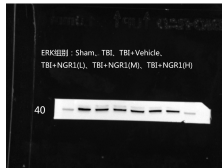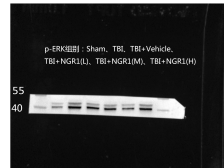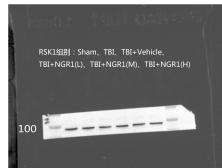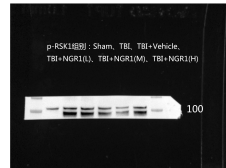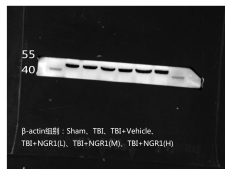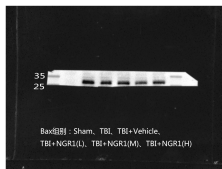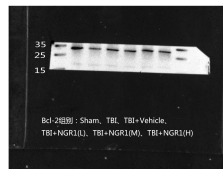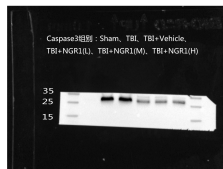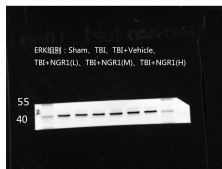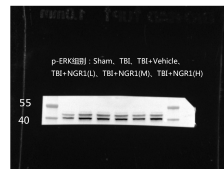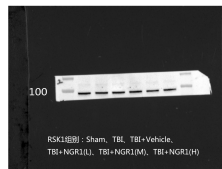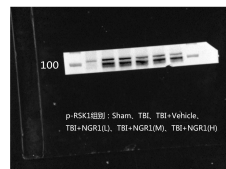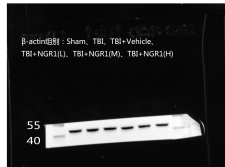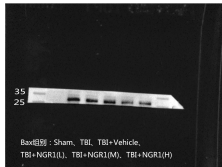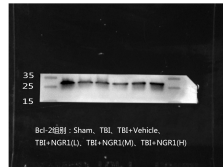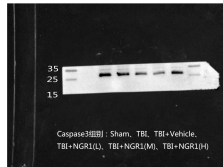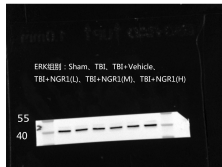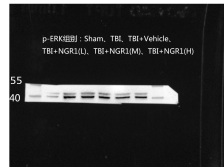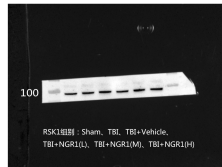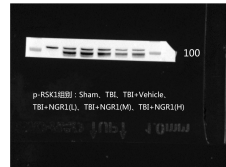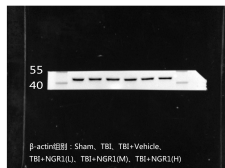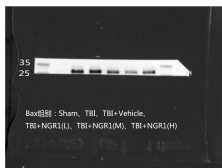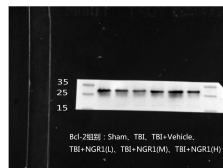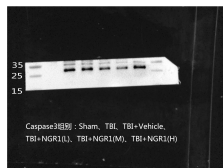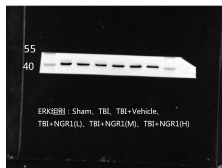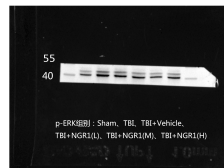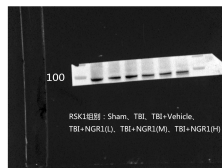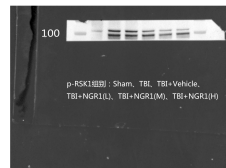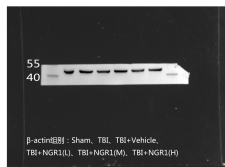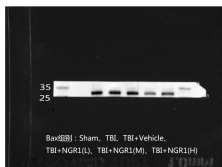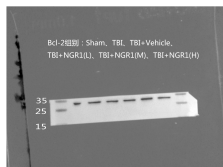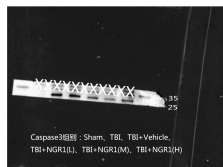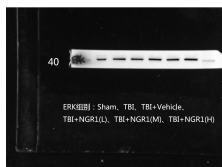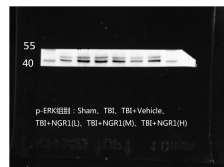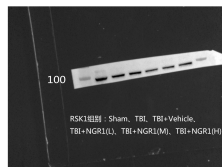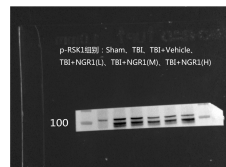

Supplement: S1 Raw images — (PDF) [file pone.0295903.s001.pdf]
