# Supplementary material for: Notoginsenoside R1 attenuates brain injury in rats with traumatic brain injury: Possible mediation of apoptosis via ERK1/2 signaling pathway
Source: PLoS One. 2023 Dec 18;18(12):e0295903. doi: 10.1371/journal.pone.0295903 (PMC10727368; doi:10.1371/journal.pone.0295903)
Supplement: Attachment — Submitted filename: Response to Reviewer.docx [file pone.0295903.s002.docx]

Dear Editors and Reviewers:

Thank you for your letter and for the reviewers’ comments concerning our manuscript entitled “Notoginsenoside R1 attenuates brain injury in rats with traumatic brain injury: possible mediation of apoptosis via ERK1/2 signaling pathway” (ID: PONE-D-23-11939). Those comments are all valuable and very helpful for revising and improving our paper, as well as the important guiding significance to our researches. We have studied comments carefully and have made correction which we hope meet with approval. Revised portion are marked in red in the paper. The main corrections in the paper and the responds to the reviewer’s comments are as flowing:

Responds to the reviewer’s comments:

Dear reviewer #1:

1.Response to comment: The protective effect of NGR1 on blood brain-barrier (BBB)still needs more tests.

Response: We understand that the immunofluorescence may better reveal the protective effect of NGR1 on blood brain-barrier (BBB). However, in the present study, we mainly focused on NGR1 can improve neuronal apoptosis in brain injury,and we think that western blotting of albumin may not be optimal, but should be sufficient to draw a conclusion that the protective effect of NGR1 on blood brain-barrier (BBB). Unfortunately, results are unavailable at this point. We agree with the reviewer, and have toned down our conclusions.

2.Response to comment: Authors also should justify whether NGR1 has the ability to cross the blood-brain barrier (BBB) or not?

Response: NGR1 belongs to saponin monomers from traditional Chinese medicine, molecular weight < 1000 KDa, we have experienced small molecules of water-soluble substances, perhaps directly through the intercellular space propagating through the blood-brain barrier. At the same time, we looked in the literature, and experiments have shown that NGR1 could actually penetrate the blood-brain barrier when given intraperitoneally. (Doi:10.3109/10715762.2014.911853).

3.Response to comment: It is suggested that authors add the importance of using effective substances derived from plants that are both affordable and effective in the area of brain trauma.

Response: It is really true as Reviewer suggested that we had update the literature on “the importance of using effective substances derived from plants” in the introduction part of the paper and enriched the introduction of the paper.

At last, thank you for your arduous work and instructive advice.

Dear reviewer #2:

Response to comment: Authors need to explain why higher dose did not work?

Response: We found that the proper dose of NGR1 was closely linked to the neuroprotective effect after a traumatic brain injury, and the intervention dose of NGR1 which was too high and too low could not have the corresponding protective effect on the brain, which was in accordance with previous literature reports, suggesting that the relation between NGR1 dose and neuroprotective effect was a U-shaped curve. Studies by Liu et al. (DOI: [10.1016/j.phymed.2021.153660](https://doi.org/10.1016/j.phymed.2021.153660)) and Zhu et al. (DOI: [10.3389/fphar.2021.615998](https://doi.org/10.3389/fphar.2021.615998)) suggest that by establishing a rat acute ischemic attack model and a medium cerebral artery occlusion/reperfusion model, both demonstrated an optimum concentration of NGR1, and zhu et al. (DOI: [10.1016/j.biopha.2021.111693](https://doi.org/10.1016/j.biopha.2021.111693)) also found that higher doses of NGR1 had no stronger neuroprotective effects. Therefore, it is assumed that the higher dose we used, 80mg/kg, could have been higher than the maximum therapeutic dose of the medication and may have no protective effect on the traumatic brain injury.

Special thanks to you for your good comments and thank you sincerely for your suggestions.

We tried our best to improve the manuscript and made some changes in the manuscript. These changes will not influence the content and framework of the paper. And here we did not list the changes but marked in red in revised paper.

We appreciate for Editors/Reviewers’ warm work earnestly, and hope that the correction will meet with approval.

Once again, thank you very much for your comments and suggestions.
