# Supplementary material for: Notoginsenoside R1 attenuates brain injury in rats with traumatic brain injury: Possible mediation of apoptosis via ERK1/2 signaling pathway
Source: PLoS One. 2023 Dec 18;18(12):e0295903. doi: 10.1371/journal.pone.0295903 (PMC10727368; doi:10.1371/journal.pone.0295903)
Supplement: Attachment — Submitted filename: Response to Reviewer.docx [file pone.0295903.s003.docx]

Dear Editors and Reviewers:

Thank you for your letter and for the reviewers’ comments concerning our manuscript entitled “Notoginsenoside R1 attenuates brain injury in rats with traumatic brain injury: possible mediation of apoptosis via ERK1/2 signaling pathway” (ID: PONE-D-23-11939). Those comments are all valuable and very helpful for revising and improving our paper, as well as the important guiding significance to our researches. We have studied comments carefully and have made correction which we hope meet with approval. Revised portion are marked in red in the paper. The main corrections in the paper and the responds to the reviewer’s comments are as flowing:

Responds to the reviewer’s comments:

Dear reviewer #1:

1.Response to comment: The protective effect of NGR1 on blood brain-barrier (BBB)still needs more tests.

Response: We acknowledge that immunofluorescence techniques could more effectively elucidate the protective effect of NGR1 on the blood-brain barrier (BBB). Nevertheless, our current research primarily concentrated on how NGR1 ameliorates neuronal apoptosis in brain injury. While we recognize that the use of western blotting for albumin may not be the most optimal method, we believe it suffices to support a preliminary conclusion regarding the protective effect of NGR1 on the BBB. Regrettably, definitive results in this regard are not yet available. In agreement with the reviewer's insights, we have accordingly moderated the assertions in our conclusions.

2.Response to comment: Authors also should justify whether NGR1 has the ability to cross the blood-brain barrier (BBB) or not?

Response: Owing to the diminutive molecular size and hydrophilic properties of phytoestrogens, compounds like NGR1, a distinct saponin present in the root of Panax notoginseng, are presumed to efficiently permeate the blood-brain barrier (Doi:10.1016/j.taap.2011.06.011). Specifically, there is a proposition of NGR1's potential modulatory role within the mammalian Central Nervous System (CNS), as suggested in recent studies (Doi:10.3109/10715762.2014.911853).

3.Response to comment: It is suggested that authors add the importance of using effective substances derived from plants that are both affordable and effective in the area of brain trauma.

Response: Indeed, as the Reviewer astutely recommended, we have updated our manuscript to incorporate an extensive review of literature emphasizing 'the importance of using effective substances derived from plants' in the introduction. This enhancement significantly enriches the context and framework of our paper, providing a more comprehensive background for our study.

At last, thank you for your arduous work and instructive advice.

Dear reviewer #2:

Response to comment: Authors need to explain why higher dose did not work?

Response: Our research indicates that the efficacy of NGR1 in providing neuroprotection following a traumatic brain injury is intricately associated with its dosage. Notably, both excessively high and low doses of NGR1 failed to offer the anticipated protective effects on the brain. This observation aligns with existing literature, suggesting a U-shaped curve relationship between NGR1 dosage and its neuroprotective impact. Studies by Liu et al. (DOI: 10.1016/j.phymed.2021.153660) and Zhu et al. (DOI: 10.3389/fphar.2021.615998), employing rat models of acute ischemic attack and middle cerebral artery occlusion/reperfusion, respectively, identified an optimal concentration range for NGR1. Furthermore, Zhu et al. (DOI: 10.1016/j.biopha.2021.111693) reported that higher doses of NGR1 did not correspond to increased neuroprotective effects. Consequently, we hypothesize that the higher dose used in our study, 80mg/kg, might have surpassed the maximal therapeutic threshold, potentially rendering it ineffective in mitigating traumatic brain injury.

Special thanks to you for your good comments and thank you sincerely for your suggestions.

We tried our best to improve the manuscript and made some changes in the manuscript. These changes will not influence the content and framework of the paper. And here we did not list the changes but marked in red in revised paper.

We appreciate for Editors/Reviewers’ warm work earnestly, and hope that the correction will meet with approval.

Once again, thank you very much for your comments and suggestions.
